# Psychiatry, subjectivity and emotion - deepening the medical model

**DOI:** 10.1192/pb.bp.113.045260

**Published:** 2014-06

**Authors:** Jessica Yakeley, Rob Hale, James Johnston, Gabriel Kirtchuk, Peter Shoenberg

**Affiliations:** 1 Tavistock and Portman NHS Foundation Trust; 2 Leeds and York Partnership NHS Foundation Trust; 3 West London Mental Health NHS Trust; 4 Camden and Islington NHS Foundation Trust

## Abstract

Morale among psychiatrists continues to be seriously challenged in the face of recruitment difficulties, unfilled posts, diagnostic controversies, service reconfigurations and public criticism of psychiatric care, in addition to other difficulties. In this article, we argue that the positivist paradigm that continues to dominate British psychiatry has led to an undervaluing of subjectivity and of the role of emotions within psychiatric training and practice. Reintegrating the subjective perspective and promoting emotional awareness and reflection may go some way towards restoring faith in the psychiatric specialty.

Psychiatry is under fire. The recent publication of DSM-5^1^ has drawn unprecedented international criticism concerning the medicalisation of normal behaviours and states of mind and accusations that a profession in pursuit of its own self-interests has become out of touch and redundant. Recruitment into psychiatric training remains a long-standing international problem,^2^ despite continuous attempts to increase its popularity among medical students. Unfilled training and consultant posts persist throughout the UK. Despite recognition of the numerous changes and challenges facing British psychiatry over the past decade or more^3^ - such as the eroding effects of perpetual National Health Service (NHS) reforms and the target culture; service reconfigurations resulting in fragmentation, splitting and loss of a developmental and attachment perspective in treatment; demotion of the role of the consultant psychiatrist in assessment, diagnosis and treatment planning yet being expected to retain responsibility for assessing and managing risk; cutting medical posts altogether under the guise of ‘new ways of working’; biogenetic reductionism and the marginalisation of psychosocial approaches; and changes in medical training - psychiatrists continue to suffer significant stress, with an increase in the proportion reporting suicidal ideation.^4^ Unsurprisingly, these discontents have an adverse impact on patients, highlighted by the recently published Schizophrenia Commission report,^5^ which makes uneasy reading in the wake of the Francis report^6^ in its description of widespread fragmentation of services, anti-therapeutic ward environments, loss of continuity of care and therapeutic relationships with trusted professionals, scarcity of psychological interventions, and denial of patients’ families as partners in care, as being all too common experiences by people with psychotic illness.

There are, of course, no easy explanations or solutions for these long-standing and complex problems, which reflect all of the complexities of working with human mental and behavioural disturbance, and we also recognise that these challenges have been and continue to be the subject of considerable informed reflection, research and debate. However, we wish to focus in this editorial on a relatively neglected or even denigrated aspect of the psychiatric endeavour that we are calling ‘affective subjectivity’. This is the awareness of and reflection on our emotional responses and their influence on our work, and the development of a capacity for self-reflection and emotional attunement with our patients. We suggest that a more widespread recognition of the potential value of affective subjectivity and its positive impact on clinical practice might go some way towards restoring faith in both the practitioners of our profession and patients.

## The dominance of positivism

Subjectivity within medicine, and psychiatry is no exception, is usually viewed as a negative quality, acknowledged as an inevitable facet of individual human experience, but considered a limitation or hindrance to the accuracy and effectiveness of the task in hand. ‘Subjectivity’ refers to the individual’s experiences, feelings, beliefs and desires, which are seen as biased, prejudiced and partisan, in contrast to ‘objectivity’ which describes a stance free from the vagaries of human perception, personal interpretation, past experiences and preconceived expectations, to reveal things as they really are. Heavily influenced by the Popperian empirical tradition in the natural sciences, British psychiatry has been suspicious of subjectivity and opted for a dominant paradigm of positivism, in which all observations are assumed to be capable of being objectively defined, validated and applied reliably.

The German philosophical school of phenomenology, developed by Karl Jaspers^7^ and adopted by British psychiatry, in itself can be seen as a method of describing, codifying and classifying the outward expressions of conscious subjective experience into an ‘atheoretical’ and therefore ‘objective’ nosological system. Although various movements - psychoanalysis, anti-psychiatry, hermeneutics - have challenged positivism in its denial of the centrality of meaning, interpretation and subjective experience within the therapeutic encounter, these approaches have tended to be marginalised within mainstream psychiatry. Similarly, the widely advocated ‘biopsychosocial approach’, promoted as a holistic way of understanding mental illness, is in practice dominated by the biological field in its greater efforts to yield ‘objective’ empirical findings than its more woolly psychological or social counterparts, illustrated by the widely publicised criticism of DSM-5’s failure to be based on ‘any objective laboratory measure’.^8^

## The fate of emotions within medical teaching

Although most would agree that elucidating the complexities of human experience cannot be achieved by brain scanning or blood tests alone, a sceptical attitude towards the value of the subjective perspective nevertheless persists, which we suggest is due, at least in part, to a (not always conscious) rejection of our own subjective responses to our work, particularly those in the emotional or affective realm.

The ambiguous position of the study of emotions within the medical discipline was identified by Balint almost half a century ago in his apt description of emotional problems in patients occupying ‘a kind of no-man’s-land: they are the province of neither the physician nor the psychiatrist’ (p. 249).^9^ Balint was critical of the at the time prevalent attitude of what he called ‘illness-centred medicine’, which was based on observations from an uninvolved ‘objective’ observer, was concerned with body parts or discrete illnesses, and encouraged an impersonal relationship with the patient, where the doctor relied on tests such as X-rays, external reports and laboratory examinations to inform diagnosis and treatment without the patient’s involvement. Balint advocated a more ‘patient-centred medicine’, which required a participating or involved observer, who thought in terms of personality difficulties and disturbed human relationships, and a relationship with the patient where information was shared. For Balint, patient-centred medicine involved the explicit study of emotions in both doctor and patient and elucidating the link between physical symptoms and emotional disturbance. Balint also highlighted the unconscious assumption a doctor makes about how a patient should be with them, which he called the doctor’s ‘apostolic function’: the doctor’s unconscious need to convert their patient to their ‘medical faith’.^10^

Since then, the patient- or person-centred approach has been widely accepted as an essential feature underpinning high-quality patient care, and it now forms a central part of undergraduate medical communication curricula. Communication skills teaching is prioritised due to increasing recognition that effective clinical communication is linked with a number of significant positive outcomes and safety for patients^11^ as well as evidence that the majority of complaints against doctors cite poor communication as the main cause of the patient’s grievance.^12^ However, the focus in teaching communication skills is on behavioural aspects such as eye contact, attentive listening, balance of open and closed questions, summarising, signposting and ‘chunking information’, whereas the role of emotional communication in the doctor-patient relationship is neglected and continues to occupy Balint’s ‘no-man’s-land’. Powerful emotions that arise within both patient and doctor may remain unspoken, minimised or completely denied.

The study of the aetiology and influence of emotional states in medical and psychiatric illness has expanded rapidly in the past half-century, aided by significant advances in the understanding of the neurobiological mechanisms of emotion. However, it appears that we may selectively use such knowledge to understand our patients, but not ourselves. We may accept that encountering the distress and suffering of medical illness may be understandably anxiety provoking and distressing for the newly qualified clinician. Doctors, however, learn from an early stage in their training to view their own emotional responses to patients with suspicion, embarrassment or outright contempt and to distance themselves from emotional contact.

Researchers have noted a worrying decline in communication skills, patient-centred attitudes and empathy in medical students as they progress through medical school.^13^ Exposed to the role models of their physician teachers, medical students may lose their idealism and wish to help others and may pick up coping mechanisms of distance and detachment at the expense of their awareness of patients’ concerns and emotions. Positive emotions such as liking or feeling attracted to patients are viewed as potentially interfering with the doctor’s ability to make objective professional judgements about diagnosis and treatment, and at worse may lead to boundary violations; experiencing negative emotions about a patient, such as feeling irritated, angry, contemptuous, disgusted or hateful may make the student feel that they are violating the respect that they are meant to show to the patient and so lead the student to denying their emotional responses completely. This has led some to propose that a wide-scale ‘professional alexithymia’ is being taught in which emotions within the doctor-patient relationship cannot be recognised, processed or regulated.^14^

Empathy has been identified as a key component of professionalism, and one of the goals in all medical education curricula in both North America and the UK is the development of empathy in learners. Thus, one of the three principle aims of the Royal College of Psychiatrists’ core curriculum is ‘to encourage students to develop appropriate attitudes necessary to respond empathically to psychological distress in all medical settings’ (p. 31).^15^ However, as with communications skills in general, the construct of empathy within medical education research has gradually shifted towards being defined as a purely cognitive process.^14^ Empathy is identified as an objective, rational, accurate, intellectual and ultimately positive process, to be distinguished from sympathy, which is regarded as emotional, self-indulgent and potentially dangerous (leading, for example, to burnout or boundary-breaking). Students are taught empathy via a set of cognitive and behavioural skills at the expense of facilitating emotional awareness and reflection. They learn that emotions are to be avoided or denied, instead of being explicitly taught how to recognise, calibrate and act on their affective responses appropriately and proportionately to clinical circumstances.

## Teaching emotional awareness in psychiatry

Tom Main^16^ differentiated between two opposing learning styles that doctors adopt towards learning about the mind and which reflect the distinction between the objective material sciences and the subjective human sciences paradigm: the diacritic (orientation towards observable data and the external world) and the coenesthetic (orientation towards feelings and the internal world). Why has psychiatric training tended to lean towards promoting the former?

The way that medicine has been traditionally taught is well described in Sinclair’s classic *Making Doctors*,^17^ in which he shows how medical students are taught to conceptualise and memorise ‘knowledge’ and ‘facts’. In this traditional model there is the potential for emotional distancing from patients by medical students as a result of negative role modelling and what has been termed ‘the hidden curriculum’.^18^ In this model, medical schools become seminaries and medical thinking becomes a form of doctrine - the ‘medical faith’ of Balint’s ‘apostolic function’.

The student of psychiatry may not be so exposed to the visible devastations of bodily illness, but to less tangible, and hence perhaps paradoxically, more anxiety-provoking, psychic disturbances. However, perhaps due to our own hardened defences, we tend to underestimate the emotional impact of encountering the more extreme states of psychotic terror, mania, sadism or obsessionality that may dominate the minds of patients with mental disorders; delusions and hallucinations of bizarre, grotesque and incomprehensible content; or the patients’ feelings of hatred and destructiveness that are enacted in bodily harm to self or others. Adopting an attitude of distance from one’s emotions here may be the reaction to underlying, less conscious fears of being overwhelmed by feelings of anxiety, terror of having no control over our emotions, and fears that we will be driven mad ourselves. Such unacknowledged affective responses may be one of the reasons why psychiatry remains an unpopular choice of career among medical students.

Within mental health services different types of supportive staff groups, such as case discussion, reflective practice and Balint groups, have been set up in recent years to encourage staff to talk about and process traumatic situations and dilemmas they encounter in their work, often led by psychodynamically informed practitioners.^19^ However, case discussion groups tend to focus on the problems of the patient from a formulation and management perspective, with little emphasis on the emotional experience of the staff.^20^ Although in reflective practice groups the clinicians’ emotional reactions to patients - referred to as ‘countertransference’ by psychotherapists - form the explicit focus of discussions, such groups are often viewed with suspicion, are rarely attended by senior members of the multidisciplinary team, and are often seen as a luxury rather than a necessity in the busy life of the psychiatric team, ward or institution.

Moreover, psychotherapists, despite being very attentive to emotional communications, may themselves be unwittingly guilty of distancing themselves from their emotional responses. The concept of countertransference has shifted from Freud’s original proposition that it represented the analyst’s unresolved emotional conflicts which posed a hindrance to therapeutic work,^21^ to one in which the patient’s contributions are emphasised as influencing or even causing the clinician’s countertransference feelings by unconscious defence mechanisms such as projection and projective identification.^22^ Although these conceptualisations have enabled countertransference to be usefully employed as a subtle tool in providing insights into the unconscious communications, modes of relating and mental state of the patient, too much of an emphasis on the patient’s contributions to the countertransference concept risks diminishing our awareness of the role of our own emotions - activated by contact with the patient, but nevertheless belonging to us and not solely ‘put into us’ by emotionally disturbed patients.

## Objectifying the subjective

Nevertheless, acknowledging the countertransference is important and our subjective emotional responses, which we may not be consciously aware of, may affect our ‘objective’ judgement, with potentially serious consequences. For example, in a study looking at the relative contribution of actuarial and emotive information in determining risk ratings of violence, Blumenthal *et al*^23^ demonstrated that experienced forensic mental health professionals, despite being well trained in the use of actuarial risk assessment tools, tended to unwittingly disregard actuarial information about the patient but were disproportionately influenced by their emotive responses to the clinical information given to them about the patient, leading them to make significant errors in risk prediction. Others have demonstrated that decisions regarding dangerousness are fundamentally based on ‘gut feelings’.^24^

Our physician colleagues may be more open to consideration of the influence of unconscious factors such as ‘intuition’ and ‘gut feelings’ on clinical decision-making. A widely cited recent study^25^ of 3890 children presenting in primary care investigated the basis and added value of clinicians’ ‘gut feelings’ and showed a significant association between such ‘intuition’ and clinical markers of serious infection. The authors defined a ‘gut feeling’ about the seriousness of illness in children as an instinctive response by clinicians to the concerns of the parents and the appearance of the children, and recommended that such responses should trigger action such as seeking a second opinion or further investigations, and that by reflecting on the genesis of their gut feeling, clinicians should be able to hone their clinical skills. It is not difficult here to substitute the term ‘countertransference’ for ‘gut feeling’ and to hypothesise that such ‘instinctive’ responses in the clinicians reflected somatic and affective manifestations of less conscious emotional communications within the doctor-patient relationship.

Such studies demonstrate that subjective affective responses may be researched by empirical or ‘objective’ methods. However, although we may need to ‘objectify the subjective’ to gain credence for the usefulness of subjectivity in our work, we may also need to ‘subjectify the objective’ in order to heal unhelpful splits in our epistemological thinking, increase our tolerance of uncertainty and ambiguities within our clinical work, and regain cognisance of the personal and the emotional within the patient and ourselves.

## Promoting affective subjectivity

There is evidence that a shift towards reintegration of the subjective perspective is occurring in recent developments within psychiatric classification, research, education and clinical practice, which challenge the polarity between mind and brain. Alternative models of the self and personality highlight the ways in which the organising of subjective experience, such as a sense of identity or affective self (that can nevertheless be objectively demonstrated via neuroimaging), may be used as a basis for classifying mental disorder.^26^ New psychosocial interventions are overtaking the ‘cul-de-sac of neurobiological approaches’ in their focus on symptom-determined areas such as paranoia or auditory hallucinations, cutting across the broader diagnostic categories of psychosis or personality disorder.^27^ Qualitative research, long established in the social sciences as a legitimate method for data collection, analysis and interpretation, but criticised for not fulfilling the objective criteria of reliability or validity satisfied by quantitative research, is enjoying a resurgence of interest in clinical and health-related research and may be more effective than quantitative approaches in exploring the complex phenomena of human behaviour.^28^

The usefulness of ‘affective subjectivity’ in its specific focus on emotions is also gaining acceptance in the wider medical as well as the psychiatric arena, due to studies showing that patients’ emotions may have a significant impact on clinical outcomes. For example, sadness and anger may amplify the experience of pain, depression may interfere with adherence to diabetic regimens, and mood states, independent from adherence, influence outcomes in medical conditions such as diabetes, myocardial infarction and cancer.^14^ However, there is also evidence that acknowledging a patient’s emotional distress is associated with increased adherence and positive outcome.^29^ Drawing on these findings, Schwartz Centre Rounds® - monthly meetings of the members of multidisciplinary medical or surgical teams designed to enhance relationships and communication by attending to psychosocial and emotional aspects of care - have become hugely popular in the USA and have been piloted in the UK since 2009 by The King’s Fund. Initial evaluations have shown positive results, including a more healthy institutional culture and greater focus on patient-centred policy and initiatives.^30^

But it is within medical and psychiatric training that we may perhaps achieve the greatest chance of ensuring that not only psychiatrists but all doctors are aware of the role of emotions in medical care. Balint groups for medical students in their first years of clinical contact with patients have now been introduced at several medical schools in the UK and plans are underway for wide-scale implementation. Other psychodynamic methods of teaching awareness of emotional communication in the doctor-patient relationship, such as medical student psychotherapy schemes, have been shown not only to enhance communication skills, but also to increase recruitment into psychiatry.^31-33^ Such methods involve learning and reflection from live emotional contacts with real patients, rather than simulated scenarios involving actors which have become the norm in communication skills teaching. The interpersonal dynamics approach,^34^ a systemised approach to utilising the subjective and interpersonal experience of staff and patients in multidisciplinary settings as a diagnostic tool, is being introduced to medical students as well as more experienced clinicians. Finally, important changes have been introduced into the core psychiatry curriculum in the shape of a new intended learning outcome (ILO) of ‘self-reflective personal development’.^35^ This defines the necessity for continuing reflective practice as a doctor and psychiatrist and the professional value of experiential emotional development in enhancing our safety and effectiveness as psychiatrists.

In promoting awareness of the subjective and emotional aspects in psychiatric training and practice, we are not trying to undermine the position of psychiatry within the natural sciences, nor erode the still fragile evidence base for the aetiology of mental disorders or efficacy of their treatment. By contrast, we suggest that a better integration of the subjective and objective paradigms will enhance creativity and innovation in psychiatric research, increase clinical benefits for patients, motivate our trainees, and contribute to restoring confidence and even ‘faith’ - albeit of a sceptical, not apostolic, nature - in the psychiatric profession.
